# Bilateral Maculopathy After Self-Inflicted Laser Pointer Injury

**DOI:** 10.3390/diagnostics15040398

**Published:** 2025-02-07

**Authors:** Bogumiła Wójcik-Niklewska, Zofia Zdort, Zofia Oliwa, Paulina Sawuła

**Affiliations:** 1 Department of Pediatric Ophthalmology, Faculty of Medical Sciences in Katowice, Medical University of Silesia, 40-055 Katowice, Poland; 2 Professor Kornel Gibiński University Hospital Center, Medical University of Silesia, 40-514 Katowice, Poland; 3 Students’ Scientific Society, Department of Ophthalmology, Faculty of Medical Sciences in Katowice, Medical University of Silesia, 40-055 Katowice, Polandzocha2002@gmail.com (Z.O.); paulina.sawula@gmail.com (P.S.)

**Keywords:** maculopathy, laser pointer, OCT, children

## Abstract

We present the case of an 11-year-old male with bilateral maculopathy caused by exposure to an astronomy laser pointer with an estimated power output of 50–100 mW. Symptoms began after exposure, and initial evaluation revealed a temporal pigment nevus in the left eye. Further examination identified bilateral retinal photoreceptor loss and subfoveal structural changes on optical coherence tomography (OCT), including reduced hyperreflectivity and retinal pigment epithelium (RPE) defects. Visual acuity was recorded as 0.6 in the right eye and 0.4 in the left eye. Laser pointer maculopathy has been increasingly reported, especially in children, raising significant public health concerns. OCT findings commonly reveal hyperreflective outer retinal changes and RPE disruptions. Prognosis varies, ranging from partial recovery to permanent visual impairment. Preventive measures, including public education and regulation of high-powered lasers, are critical to mitigating this avoidable cause of retinal injury. This case highlights the importance of prompt diagnosis, imaging, and awareness to address the growing prevalence of laser-induced ocular injuries.

**Figure 1:**
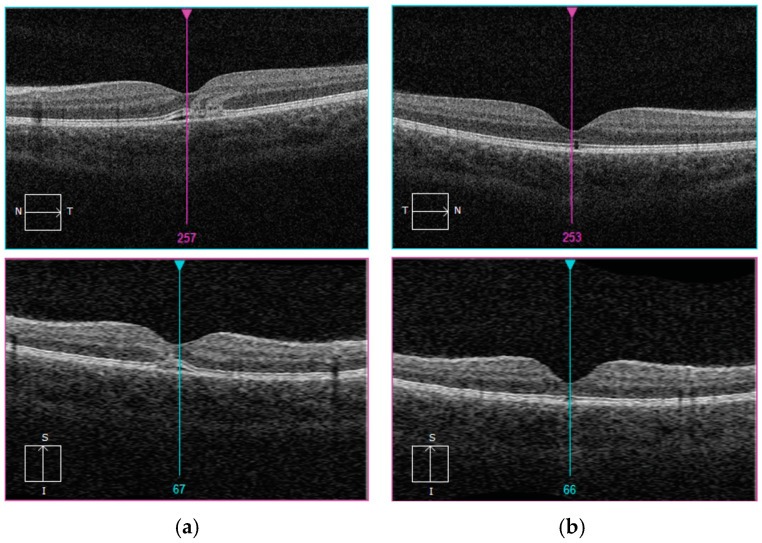
(**a**,**b**) Initial evaluation in the emergency department revealed a temporal pigment nevus in the fundus of the left eye, prompting referral to our pediatric ophthalmology clinic. Upon further assessment in our department, the patient was diagnosed with bilateral maculopathy secondary to laser exposure, accompanied by a loss of retinal photoreceptors in both eyes. At presentation, visual acuity measured 0.6 in the right eye and 0.4 in the left eye. Optical coherence tomography (OCT) imaging of both eyes was performed at the time of admission and discharge (hyporeflective space in the outer layers of the retina and disturbances of the photoreceptors layer (**a**) and, after typical treatment, at the end of the observation period, defect of ellipsoid zone of the photoreceptors and retinal pigment epithelium (**b**). An 11-year-old male who presented to the emergency department with visual disturbances occurring two days after exposure to an astronomy laser pointer. The power output of such laser devices typically ranges between 50 mW and 100 mW. It is presumed that the laser beam reflected off an unidentified surface, causing bilateral ocular damage. Laser pointer maculopathy, resulting from direct exposure of the retina to handheld laser beams, has been increasingly reported in pediatric populations, reflecting a growing public health concern [1]. Although many laser pointers, including Class 3R lasers (<5 mW), are labeled as “safe”, evidence suggests that even these devices can induce significant retinal damage, particularly in children who may unintentionally fixate on the beam [2]. Optical coherence tomography (OCT) imaging in such cases frequently reveals characteristic hyperreflective changes in the outer retinal layers and disruptions of the retinal pigment epithelium (RPE), particularly at the fovea [3,4]. Clinical presentations typically include central scotomas, metamorphopsia, or blurred vision, with symptoms manifesting acutely after exposure [2,4]. The prognosis varies significantly. While some cases show partial recovery of visual acuity, others result in permanent deficits, often correlating with persistent OCT abnormalities such as RPE irregularities and photoreceptor damage [4]. A large UK case series highlighted the increased prevalence of these injuries in children, underscoring the need for preventive measures and stricter regulation of high-powered laser devices [2]. Our case mirrors these findings, demonstrating both the structural damage visible on OCT and the functional visual impairments described in the prior literature. Current management strategies prioritize prompt ophthalmological assessment and multimodal imaging, though no standardized treatment protocol exists [3]. Preventive efforts, including public education about the risks of laser pointers and behavioral interventions to discourage misuse, remain critical in mitigating this avoidable form of retinal injury [4]. As the incidence of laser pointer-induced retinal injuries continues to rise, it is imperative to address this avoidable cause of visual impairment. Comprehensive efforts encompassing enhanced device regulation, targeted public awareness campaigns, and further research into effective therapeutic strategies are critical to mitigating this growing concern [2,4].

## Data Availability

All relevant data are within the manuscript.
